# Cardiovascular Diseases and Women: Knowledge, Attitudes, and Behavior in the General Population in Italy

**DOI:** 10.1155/2015/324692

**Published:** 2015-01-28

**Authors:** Luisa Maria Roberta Tedesco, Gabriella Di Giuseppe, Francesco Napolitano, Italo Francesco Angelillo

**Affiliations:** Department of Experimental Medicine, Second University of Naples, Via Luciano Armanni 5, 80138 Naples, Italy

## Abstract

*Background*. The objectives of the study were to document knowledge, attitudes, and behaviors of women regarding cardiovascular diseases (CVDs) and the determinants associated. *Materials and Methods*. The cross-sectional survey was conducted among a random sample of 830 women older than 18 years from the general population in Italy. *Results*. Almost all participants reported having heard about CVDs, and among them 89.4% and 74.7% identified smoking and high cholesterol level as risk factors. Only 26.5% identified the main CVDs risk factors. Women more knowledgeable were married and better educated and self-perceived a worse health status. Only 23% knew the main CVDs preventive measures and this knowledge was significantly higher in women who are unemployed, who are more educated, who have received information about CVDs from physicians, and who know the main risk factors. Respondents with lower education, those with at least three children, those who self-perceived a worse health status, and those who need information were most likely to have a positive attitude toward the perceived risk of developing CVDs. Women with two or three children or more were at high risk profiles 49% and 56% lower than women with one child. *Conclusions*. Educational programs are needed among women as support to improve knowledge and appropriate behavior about CVDs.

## 1. Introduction

It is well established that cardiovascular diseases (CVDs) continue to be the leading cause of morbidity and mortality in the general population of many developed and developing countries [1–5] and several modifiable lifestyle risk factors, including unhealthy diet, physical inactivity, cigarettes smoking, alcohol consumption, and body mass, are at an alarming level. These risk factors have been identified which act in variable combinations with other genetic, physiologic, and environmental components to influence the incidence of CVDs or affect the short/long-term outcome of the disease. There is also substantial evidence to indicate the importance of primary and secondary prevention of CVDs and early identification designed to focus on these risk factors. Indeed, their rigorous control is widely recommended as an essential aspect of public health measures in order to effectively have a substantial overall impact on risk of CVDs through the practice of healthy lifestyle risk factors and related health behaviors, quality of life, and mortality for the majority of cardiovascular burdens [6–8].

It is important to underline that specifically adequate knowledge, positive awareness, and appropriate health promotion behaviors regarding CVDs in the general population are crucial given their potential impact on the adherence to programs and interventions for reducing the incidence of CVDs. The level of knowledge and the behaviors toward CVDs among the general population during the recent years have been researched and the literature concerning these issues is abundant. However, to date very little attention, to the best of our knowledge, has been given to the issue of women's knowledge, attitudes, and behaviors toward CVDs [9–12]. Therefore, in this current investigation, conducted among a large sample of women from the general population in Italy, the primary objective was to document the level of knowledge, the attitudes, and the reported behaviors regarding CVDs and the second objective was to examine the extent to which determinants are associated with these outcomes.

## 2. Materials and Methods

The cross-sectional survey took place between October and December 2012 targeting a population group of women older than 18 years living in the areas of Naples and Salerno, Italy.

Potential participants were the mothers of children aged 3–18 years randomly recruited from five public schools randomly selected from the list of different kind of schools in the areas. The manager of each selected school was contacted and after their approval, the research team distributed to all pupils an envelope addressed to their mothers with an invitation letter, a questionnaire, an informed consent form, and a self-addressed envelope for returning the questionnaire. The invitation letter included a description of the study, information about anonymity of participation, and the noncompulsory nature of information given. Those who chose to participate gave their written informed consent before their interview.

An overall prevalence of women who have heard about CVDs was expected to be close to 50%, assuming a confidence interval of 95%, a maximum error of 5%, and a conservatively estimated design effect of two, and considering a response rate of 70%, the sample size was calculated to be at least of 476 women to estimate the main objectives of the study.

Data was collected using a semistructured self-administered anonymous questionnaire that was pilot-tested on 20 women. The instrument included questions grouped into 5 areas: (1) sociodemographics and health history; (2) knowledge of the CVDs' risk factors and preventive measures; (3) attitudes toward CVDs by measuring their perception of risk, the possibility of having a CVD in their life, the possibility of preventing CVDs, and the importance of a prudent diet and of adequate sport activity to prevent CVDs; (4) lifestyle, smoking habit, physical activity, alcohol consumption, and diet: with regard to health-promoting behavior, respondents were asked to indicate whether or not they have checked in the past year glucose, blood pressure, and cholesterol; (5) sources of information about CVDs.

The Ethics Committee of the Second University of Naples approved the study.

### 2.1. Statistical Analysis

The statistical analysis has been conducted in two stages. First, Student's *t*-test for independent samples and chi-square test have been conducted to determine their association with the outcomes of interest and the variables found to be associated at the *P* value ≤ 0.25 level were introduced into multivariate regression models. Second, multivariate logistic and linear regression have been performed to investigate independent characteristics associated with the dichotomous and continuous outcomes of interest. Four models have been constructed: adequate knowledge regarding the main CVDs risk factors (Model 1), adequate knowledge regarding the preventive measures of CVDs (Model 2), perceived risk of developing CVDs (Model 3), and women's high risk profile (Model 4). A stepwise backward elimination process has been used and the final models only included variables providing a significant explanation of outcomes, in which the criterion for entering and exiting in the model was, respectively, of *P* value > 0.2 and *P* value < 0.4. In all models, the following independent variables were included: age, marital status, educational level, occupation, number of children, having at least one chronic disease, self-perceived health status, physician as source of information about CVDs, and needs of additional information about CVDs; the variables knowledge of the CVDs' main risk factors (smoking, high cholesterol level, diabetes, high blood pressure, and fatty diet) and knowledge of the CVDs' preventive measures (quitting smoking, engaging in physical exercise, lowering diet salt, having blood pressure and blood cholesterol level checked, being in shape, and eating fruit and vegetables) were also included in Models 2 and 3; the variables knowledge that sedentary life is a CVDs' risk factor, knowledge that physical exercise helps to prevent CVDs, knowledge that no cigarette smoking helps to prevent CVDs, and worry about developing CVDs were also included in Model 4.

In the logistic regression models, odds ratios (ORs) and their 95% confidence intervals (CIs) were calculated. Standardized regression coefficients (*β*) were presented in the linear regression model. All statistical tests were two-tailed and differences were considered to be statistically significant at a *P* value ≤ 0.05. All analyses were conducted using Stata version 10.1 statistical software [13].

## 3. Results

A total of 922 women were recruited and invited to fill out the self-administered questionnaire; 830 agreed to participate in the study for a response rate of 90%.  Table 1 provides an overview of the respondents sociodemographic and general characteristics. The age of the women ranged from 21 to 67 years, the average age was 38 years, almost all were married (91.7%), about one in five had a college degree, more than three-quarters had more than one son, almost half reported employment, and only 1.1% reported a personal history of CVD.

Table 2 describes the percentages of correct response to each individual item regarding the knowledge about CVDs. Almost all of the participants (96.1%) reported having heard about CVDs, and among them, respectively, 89.4% and 74.7% correctly identified smoking and high cholesterol level as risk factors for CVDs, while only 46.3% and 31.5% indicated diabetes and familiarity. Overall, only 26.5% of the respondents were able to correctly identify the main CVDs risk factors.  Table 3 shows the odds ratios with logistic regression for the predictors of the different outcomes of interest. The results of the multiple logistic regression analysis showed that four independent variables were identified as statistically associated with the correct knowledge of the CVDs main risk factors. Married participants (OR = 2.53; 95% CI 1.18–5.45) and those who self-perceived a worse health status (OR = 0.82; 95% CI 0.75–0.90) were more likely to have this knowledge. Women with a high school (OR = 0.57; 95% CI 0.36–0.89) or not higher than middle school level of education (OR = 0.2; 95% CI 0.1–0.37), compared with those with a college degree or higher qualification, and those who have three or more children (OR = 0.35; 95% CI 0.22–0.57), compared with those with just one child, were less knowledgeable (Model 1 in Table 3). When asked about the preventive measures for CVDs, the vast majority correctly indicated quitting smoking (87%), engaging in physical activity (71.8%), and losing weight (71%) as preventive measures, but only half identified fruit and vegetables intake. Overall, only 23% of the group knew the main CVDs preventive measures. Multivariate analysis was done using logistic regression model to investigate the predictors of correct knowledge of the preventive actions toward CVDs. The result of this analysis revealed that women who have received information about CVDs from physicians were two times more likely to know the preventive actions compared to those who have used other source(s) (OR = 2.01; 95% CI 1.38–2.93). Correct knowledge of the main risk factors of CVDs was also a significant predictor, since women who have this knowledge were almost seven times more likely to know the preventive actions compared to the less knowledgeable group (OR = 6.74; 95% CI 4.53–10.02). Moreover, those occupied (OR = 0.63; 95% CI 0.41–0.98) and with a middle school or lower education (OR = 0.49; 95% CI 0.26–0.95), compared with those who had at least a college degree, were less knowledgeable (Model 2 in Table 3).

The data concerning the attitudes toward CVDs in the study population showed that more than half responded that is possible to prevent CVDs and, respectively, 83.5% and 81.4% responded that healthy diet and sport activity help to prevent CVDs. Participants were also asked to rate their perceived risk of developing CVDs and the mean score was 6 indicating a low level of risk perception, with only 9.9% asserting a high degree of concern by answering “10.” The multivariate linear regression analysis identified that the most important variables independently associated with the level of risk perception were respondents' educational level, number of children, needs of additional information toward CVDs, and the self-perceived health status. Respondents with a lower educational level, those who have three or more children, those who need more information about CVDs, and those who self-perceived a worse health status were most likely to have a positive attitude toward the perceived risk of developing CVDs (Model 3 in Table 3).

Very few women (24.9%) have a high risk profile for developing CVDs. Among the recommended self-care behaviors, drug adherence (78.4%) and dietary intake (57.7%) were those most practiced. On the other hand, moderate physical exercise was the least practiced recommended behavior and 27.9% of the total sample was current smokers (Table 4). Multiple logistic regression analysis was used to determine the extent to which a high risk profile of the study population varied with the several variables. The results indicated that only one variable was statistically associated with the outcome of interest. The odds of respondents with two or at least three children were, respectively, at high risk profiles 49% (OR = 0.51; 95% CI 0.33–0.79) and 56% (OR = 0.44; 95% CI 0.26–0.74) lower than women with only one child (Model 4 in Table 3).

In terms of information, participants were asked to indicate their sources of CVDs information from a list of sources. The most frequently chosen category was radio/television (53.7%), followed by physicians (46.1%) and family (34.8%). 74.1% of the respondents indicated that they would like more information about CVDs.

## 4. Discussion

This study regarding the knowledge, attitudes, and behaviors towards CVDs, the first large population-based study among women in Italy, provided information for educators and policy makers for implementing preventive campaigns in this population.

This sample was not very erudite although almost all have heard about CVDs (96.1%), with overall low levels of knowledge of the CVDs' main risk factors and preventive actions. However, adequate levels have been observed for some questions. For example, 89.4% and 74.7% identified smoking and high cholesterol level as CVDs' risk factors, but less than half identified diabetes. Quitting cigarette smoking (87%), engaging in physical exercise (71.8%), losing weight (71%), and reducing dietary salt intake (67.5%) have been identified as measures for reducing the risk of getting CVDs. These results may be compared to previous findings. In the United States, adults aged 18–26 years identified engaging in physical exercise (93.3%), losing weight (87.5%), reducing sodium or salt in diet (86.5%), and quitting smoking (84.6%) as preventive measures of getting heart disease [11]. In American Indian women with previous gestational diabetes, the majority was aware of the roles of activity, diet, cholesterol, and family history related to risk of CVD [12]. In the French West Indies, women identified “physical exercise or sport activity” (47%) as precaution to avoid heart disease, followed by “eat less fat” (42%), “drink less alcohol” (26%), and “do not smoke” (24%) [9].

In this study, the majority of the women reported appropriate conduct and very few have a high risk profile for developing CVDs. Including smoking and lack of physical exercise in a high risk profile, only one-fourth have this profile. Among the recommended self-care behaviors, drug adherence and dietary intake were most practiced, whereas moderate physical exercise was the least recommended behavior practiced and approximately one-third were current smokers. Women with one child were at a lower risk profile compared with those with more than one. Good attitudes did not lead to better self-reported practices and this indicates the need to promote the utilization of strategies through encouragement of compliance with prevention practices, which will result in more efficient CVDs control. This highlights that to bring behavioral change implementing a multifactorial approach is needed, which also targets other important facets, such as the sustainability of CVDs control programs.

Television and physicians were the major sources of information. Therefore, scientific TV programs about CVDs are in great need to improve the knowledge. Women who had received information from physicians were more likely to know the preventive strategies toward CVDs. This finding is consistent with previous studies in the same area confirming that exposure to health information results in better knowledge [14, 15]. Thus, physicians, especially general practitioners, ought to strengthen health education and appropriate surveillance of the population. The important role of the sources of information is also supported by the finding that women who need information have a positive attitude toward the perceived risk of contracting CVDs.

Identifying the characteristics of women who have and do not have adequate level of knowledge or positive attitudes can help to target efforts. Married women and those more educated were more likely to know the risk factors. The positive effect of the education on the knowledge is consistent with previous research [11, 16, 17]. Moreover, those who self-perceived a worse health status were less knowledgeable and a possible explanation is that such perception may cause worries of developing CVDs with more attention to these diseases with consequent higher knowledge regarding the risk factors. The women's attitude indicated that those with lower education, those with more than two children, and those who needed information about CVDs were more likely to have a high self-risk perception of developing CVDs. The reasons for the finding regarding the number of children are unclear; possibly women with just one child are more concerned about health care issues as they feel responsible for his or her health and, therefore, for her family.

In interpreting the results, there are potential limitations that might impact upon the conclusions drawn. First, this study has a cross-sectional design and the ability to establish the directionality in the relationship between the dependent and independent variables cannot be verified. Second, the survey used self-reported information, which may overestimate or underestimate the actual attitude and practices. This risk was minimized by administering written surveys and the absence of identifying data. Third, the selection of mothers of children aged 3–18 years may result in a sample of a lower average age, risk profile, and cardiovascular mortality than the general population [18]. Therefore, it may not be representative of the whole Italian women population. Despite these limitations, the strengths of this study were the fact that the sample was large and properly selected and the high response rate.

## 5. Conclusions

This large epidemiological study provided important data concerning knowledge, attitudes, and reported behaviors regarding the CVDs among a population of women. From a public health perspective, it is imperative that health educational programs are needed as support to improve the level of knowledge and appropriate behavior about CVDs in the general population.

## Figures and Tables

**Table 1:** Self-reported characteristics and health status of participants in the study.

	*N*	%
Age (years)	38 ± 6.1 (21–67)^*^	
18–30	65	7.9
31–35	245	29.6
36–40	292	35.3
41–45	145	17.4
>45	81	9.8
Body mass index (BMI)	25.1 ± 4.5 (16.8–52.7)^*^	
Underweight	8	1.1
Normal	87	58.7
Overweight	226	27.2
Obese	109	13
Marital status		
Married	761	91.7
Other	69	8.3
Educational level		
No formal education, elementary or middle school	236	28.6
High school	433	52.6
College degree or higher	155	18.8
Occupation		
Employed	438	55.9
Unemployed	346	44.1
Number of children		
1	129	16.4
2	447	56.7
≥3	212	26.9
Personal history of CVDs^†^		
Yes	9	1.1
No	821	98.9
Personal history of hypertension		
Yes	69	8.3
No	761	91.7
Personal history of diabetes mellitus		
Yes	8	1
No	822	99
Personal history of high cholesterol levels		
Yes	33	4
No	797	96
Menopause		
Yes	42	5.1
No	788	94.9
Hormone replacement therapy^‡^		
Yes	13	31
No	29	69
Self-perceived health status	7.4 ± 1.9 (1–10)^*^	

Number for each item may not add up to total number of study population due to missing value.

^*^Mean ± standard deviation (range).

^†^Cardiovascular diseases.

^‡^Only for women who have already reached menopause.

**Table 2:** Women's knowledge about main risk factors and preventive measures for cardiovascular diseases (CVDs).

	*N*	%
Have heard about CVDs		
No	32	3.9
Yes	789	96.1

	Correct answer	
	*N*	%

Risk factors^*^		
Smoking	715	89.4
High blood pressure	601	75.3
High cholesterol level	592	74.7
High weight	560	70.2
High salt diet	489	61.4
Sedentariness	378	47.3
Diabetes mellitus	361	46.3
Familiarity	278	31.5
Preventive measures for CVDs		
Quit smoking	694	87
Have cholesterol level checked	594	75.4
Physical activity	568	71.8
Lose weight	558	71
Have blood pressure checked	547	69.9
Reducing diet salt	532	67.5
Fruit and vegetables intake	396	50.1

^*^Only for those who reported that they have heard about CVDs.

**Table 3:** Multivariate logistic and linear regression analyses indicating associations between several variables and the different outcomes regarding cardiovascular diseases (CVDs).

Variable	OR	SE	95% CI	*P* value
Model 1: knowledge of the CVDs' main risk factors (smoking, cholesterol, diabetes, hypertension, fatty diet)				
Log likelihood = −396.29, *χ* ^2^ = 117.52 (6 df), *P* < 0.0001				
Self-perceived health status	0.82	0.03	0.75–0.90	<0.001
Number of children				
One	1^*^			
Three or more	0.35	0.08	0.22–0.57	<0.001
Educational level				
College degree or higher	1^*^			
No formal education/elementary/middle school	0.2	0.06	0.1–0.37	<0.001
High school	0.57	0.13	0.36–0.89	0.014
Marital status	2.53	0.99	1.18–5.45	0.017
Occupation	0.82	0.16	0.55–1.21	0.323

Variable	OR	SE	95% CI	*P* value

Model 2: knowledge of the CVDs' main preventive measures				
Log likelihood = −348.11, *χ* ^2^ = 157.48 (8 df), *P* < 0.0001				
Physician as source of information	2.01	0.39	1.38–2.93	<0.001
Knowledge of main CVDs' risk factors	6.74	1.36	4.53–10.02	<0.001
Educational level				
College degree or higher	1^*^			
No formal education/elementary/middle school	0.49	0.16	0.26–0.95	0.035
High school	0.68	0.17	0.41–1.11	0.127
Occupation	0.63	0.14	0.41–0.98	0.042
Self-perceived health status	1.10	0.06	0.99–1.22	0.059
Marital status	1.76	0.74	0.77–4.00	0.178
Age	1.01	0.01	0.98–1.04	0.289

Variable	Coeff.	SE	*t*	*P* value

Model 3: worries about developing CVDs				
*F*(9.733) = 6.72, *P* < 0.0001, *R* ^2^ = 0.07%, adjusted *R* ^2^ = 0.06%				
Need of additional information toward CVDs	0.72	0.2	3.57	<0.001
Number of children				
One^*^				
Two	0.43	0.23	1.88	0.061
Three or more	0.93	0.27	3.42	<0.001
Self-perceived health status	−0.14	0.04	−2.97	0.003
Educational level				
College degree or higher^*^				
No formal education/elementary/middle school	0.5	0.2	2.46	0.014
Having at least one chronic disease (CVDs, diabetes, obesity, hypertension)	0.31	0.17	1.83	0.067
Physician as source of information	0.28	0.16	1.67	0.096
Knowledge of the CVDs' main risk factors (smoking, cholesterol, diabetes, hypertension, fatty diet)	−0.29	0.19	−1.52	0.129
Age	0.01	0.01	1.11	0.265

Variable	OR	SE	95% CI	*P* value

Model 4: women's high risk profile				
Log likelihood = −419.71, *χ* ^2^ = 35.10 (9 df), *P* = 0.0001				
Number of children				
One	1^*^			
Two	0.51	0.11	0.33–0.79	0.003
Three or more	0.44	0.11	0.26–0.74	0.002
Educational level				
College degree or higher	1^*^			
High school	0.66	0.15	0.42–1.02	0.064
No formal education/elementary/middle school	1.33	0.34	0.81–2.20	0.252
Knowledge of no smoking as a CVDs' preventive measure	1.56	0.53	0.80–3.06	0.187
Knowledge of smoking as a CVDs' risk factor	1.57	0.61	0.73–3.38	0.240
Self-perceived health status	0.95	0.04	0.87–1.04	0.308
Knowledge of physical activity as a CVDs' preventive measure	1.22	0.25	0.82–1.83	0.311
Marital status	0.75	0.23	0.41–1.36	0.349

^*^Reference category.

**Table 4:** Questions assessing behavior toward cardiovascular diseases (CVDs).

	*N*	%
Have ever smoked		
Yes	300	36.1
No	530	63.9
Current smoker		
Yes	232	27.9
No	598	72.1
Have drunk in the last 30 days		
Yes	249	30.1
No	579	69.9
Moderate physical exercise		
Yes	211	26.6
No	581	73.4
Vigorous physical exercise		
Yes	122	15
No	690	85

Number for each item may not add up to total number of study population due to missing value.
